# Capacity indicators for disaster preparedness in hospitals within Nairobi County, Kenya

**DOI:** 10.11604/pamj.2014.18.349.3586

**Published:** 2014-08-29

**Authors:** Cynthia Nekesa Simiyu, George Odhiambo-Otieno, Dominic Okero

**Affiliations:** 1 Department of Health Systems Management, Kenya Methodist University, Nairobi County, Kenya

**Keywords:** Capacities, disaster preparedness, health Systems, hospitals, Nairobi County

## Abstract

The goal of this study was to assess hospital capacity for disaster preparedness within Nairobi County. This information would be valuable to institutional strategists to resolve weaknesses and reinforce strengths in hospital capacity hence ensure efficient and effective service delivery during disasters. Analytical cross-sectional research design was used. Indicator variables for capacity were hospital equipment, hospital infrastructure, surrounding hospital environment, training, drills, staff knowledge and staff capabilities. Thirty two hospitals were studied of which nine of them were public hospitals. Data analysis was done using SPSS and presented in the form of frequency tables at p < 0.05. Study results indicated that hospital capacity to disaster preparedness in Nairobi County existed in 22 (68.88%) hospitals, in 6 (64.95%) public hospitals and 16 (69.64%) private hospitals. The difference in capacity between public and private hospitals within the County was less than 5%. This showed that both public and private hospitals were relatively at par, with regard to the capacity to handle disaster cases. Study findings also revealed that the surrounding hospital environment was the most highly rated indicator while inter hospital training and drills were the least rated. Although existent in hospitals within Nairobi County, for maximum hospital capacity and disaster preparedness within Nairobi County to be achieved, the existent gap in inter hospital training and inter hospital drills, both of which fall under the finance health systems pillar, required addressing.

## Introduction

The World Health Organization (WHO) defined Health Systems (HS) as all organizations, institutions and resources that are devoted to producing health actions [1]. Increasing hospital resilience through disaster preparedness enables timely, efficient and effective service delivery during disaster cases and this contributes to Health Systems Strengthening (HSS). According to the United Nations (UN) and the International Strategy for Disaster Reduction (ISDR), capacity is defined as a combination of all the strengths and resources available within a community, society or organization that can reduce the level of risk, or the effects of a disaster and may include physical, institutional, social or economic means as well as skilled personal or collective attributes [2]. Pillars in Health Systems (HS) that result in HSS include service delivery, health workforce, information, financing, stewardship, medical products, vaccines and technologies [3]. The physical aspect of capacity is linked to the health workforce HS pillar and they should be skilled and knowledgeable. The economical aspect of capacity comprises the finance HS pillar and this facilitates disaster preparedness training programs and drills. Medical products, vaccines and technology HS pillar comprise the institutional aspect of capacity which is basically HS equipment and HS infrastructure. The social aspect of capacity refers to the HS surroundings. The social aspect of capacity has a bearing on the information HS pillar. Nairobi County joined the World Disaster Risk Reduction campaign where one theme was ‘hospitals safe from disasters’ [4]. This theme aimed at increasing hospital resilience to disasters through strengthening hospital capacity and policies. However, no studies had been initiated to determine existence of disaster preparedness hospital capacities within Nairobi County which would be a starting point in identifying strengths, gaps and opportunities for HSS. It would also be informative to discover if any differences in disaster preparedness capacities existed between public and private hospitals within the County. This study aimed at establishing existence of capacities for disaster preparedness in hospitals within Nairobi County.

## Patient and observation

Analytical cross sectional research design was used. Study variables were hospital equipment, hospital infrastructure, the surrounding hospital environment, training, drills, staff knowledge and staff capability. The hypothesis in this study was ‘Mean score capacity does not differ significantly between Public and Private Hospitals in Nairobi County, Kenya‘. The study setting was hospitals in Nairobi County, Kenya. 32 hospitals cooperated during the research, 9 of them public and 23 private hospitals. Study respondents were the hospital workforce categorised as either clinical or administrative staffs. Clinical staffs comprised those whose primary responsibility was to save lives and doctors and nurses were the main respondents. Administrative staff comprised those responsible for coordinating the HS processes within the hospitals. They comprised; the Chief Medical Officer, Financial Controller, the Disaster Management Committee Chair, the Communications Department Chair and the Human Resources Director.

Based on information by Ndetei, Khasakhala and Omolo, [5], the number of administrative and clinical staffs in Nairobi County was obtained to be 18707. Slovin's formula for random sampling [6] was used to get the representative sample at 95% level of confidence. The estimated sample size obtained was 391.6 and this was adjusted to 400. Upon field work, 263 respondents participated which was 65.75% participation. Research instruments were questionnaires, participant observation checklist and key informant interviews. Ethical consideration was obtained from the National Council of Science and Technology, Kenya and the hospital ethical committees. A confidentiality clause was also included on the questionnaire form. Field work was executed between the months of June to August 2012. Research assistants took time to fill the observers’ checklist and used the key informant interview form to interview administrative staff. Questionnaires were self administered on the clinical staff. Statistical significance was considered at p value < 0.05. Data obtained after fieldwork was filtered, coded and keyed into Statistical Packages for Social Scientists (SPSS) version 10. A detailed description of study results obtained is tabulated in (Table 1).

**Table 1:** Summary of mean percentages obtained for various capacity indicators

Hospital capacity indicator measure	All hospitals (32)		Private Hospitals (23)		Public hospitals (9)	
	Number	%Mean	Number	%	Number	%
Surrounding hospital environment	29	90.00%	21	91.32%	8	86.68%
Training (intra-hospital)	28	88.90%	20	88.80%	8	89.50%
Staff knowledge	28	87.35%	20	87.80%	8	85.45%
Hospital equipment	24	75.40%	17	75.60%	7	74.50%
Staff capabilities	23	72.75%	17	73.80%	6	68.45%
Hospital infrastructure	21	67.10%	15	65.60%	6	64.20%
Drills (intra-hospital)	21	66.70%	17	72.50%	4	42.10%
Training (inter-hospital)	17	54.50%	13	57.50%	4	42.10%
Drills (inter-hospital)	6	17.20%	3	13.80%	3	31.60%
Mean	22	68.88%	16	69.64%	6	64.95%

## Discussion

The capacity of hospitals to efficiently manage disasters within Nairobi County was established to exist in 22 (68.88%) out of the 32 hospitals. This capacity was slightly less in 6 (64.95%) out of 9 public hospitals compared to 16 (69.64%) out of 23 private hospitals. Capacity was good (60-95%) with regard to hospital equipment, hospital infrastructure, hospital surroundings, staff knowledge and staff capability indicators. However, inter hospital drills and training was low (less than 60%), reducing the effectiveness of disaster preparedness for HSS. The surrounding hospital environment, intra-hospital training and staff knowledge scored highly for capacity indicators at 90.00%, 88.90%and 87.35% respectively. Hospital equipment and staff capabilities scored relatively well at 75.40% and 72.75% respectively. The score for hospital equipment from this study agreed with study findings of Kaji and Lewis. [7] in Los Angeles County who found adequate hospital equipment for disaster preparedness. These results however were slightly inferior to findings from Poland [8] where in addition to availability of hospital equipment, they had surveillance systems for disaster preparedness which should be a recommendation for hospital equipment in Nairobi County with regard to disaster preparedness.

Staff capability referred to an individual's ‘ability’ to take action using their ‘capacity’. Staff ‘ability’ was measured by checking whether the respondents were conversant with their individual roles in case of a disaster and this recorded high scores at 88.9%. Staff ‘capacity’ was measured by the respondents’ view regarding the resources available for disaster preparedness within the hospitals and scores obtained were not so highly rated at 56.6%. Since capacity comprises human (staff), equipment and infrastructure, the respondents seemed to fault equipment and infrastructure, with regard to staff capability. Results showed a marked difference between staff ‘capacity’ in private versus private hospitals: 94 (58.8%) clinical respondents and 19 (47.4%) clinical respondents respectively. This indicated that respondents from private hospitals were more confident on the existent hospital resources for disaster preparedness compared to those from public hospitals, a factor that is recommended for further research.

To assess hospital infrastructure, data was collected on hospital building layout, structural integrity of buildings, power back up, evacuation plan, emergency exits, fire detecting equipment (smoke detectors, fire alarms) and firefighting equipment (halon fire extinguishers and hose reels). Hose reels and structural assessment of building measures were lowly rated for hospital infrastructure indicator. Hose reels seemed to be replaced by the high availability of halon fire extinguishers. Hospital infrastructure and intra hospital drills were rated at 67.10% and 66.70% respectively. The obtained rating of hospital infrastructure (67.10%) sheds some light as to why the ‘capacity’ aspect of staff capability readings were not so highly rated (56.6%), as explained in the preceding paragraph. Inter-hospital training and inter-hospital drills scored poorly at 54.50% and 17.20% respectively. Results by Kaji et al. showed that interagency training was not integrated within Los Angeles County which he observed ‘limited surge capacity’ [7]. Results obtained by Gupta et al. on hospitals in India showed neither ‘external coordination nor conduction of joint disaster drills amongst the hospitals’ that contributed to ‘low awareness on disaster preparedness’ hence reducing hospital capacity [9]. The low scoring of inter-hospital drills obtained in this study indicated it as an urgent gap to be filled for optimal hospital capacity and disaster preparedness. Training and drills are classified under the finance HS pillar and the low rating from this study was an indication that there was a high probability of inadequate financing towards training and drills.

The hypothesis in this study was that ‘Mean score capacity does not differ significantly between Public and Private Hospitals in Nairobi County, Kenya’. SPSS was used to run a two-tailed Pearson's chi-square test using data obtained on mean percentages of capacity indicators categorised into private and public hospitals from (Table 1). The Pearson's chi-square obtained was X2 (56, n = 9) =63, p = 0.243 as shown in (Table 2). From the results p = 0.243 was greater than p = 0.05. Therefore, results failed to indicate a significant difference and the hypothesis ‘mean score for capacity does not significantly differ between Public and Private Hospitals in Nairobi County, Kenya’ was retained. These results agreed with study results where the difference between public and private hospitals was less than 5% hence insignificant.

**Table 2:** SPSS Results for Mean Score for hospital capacity

	Value	Df	Asymp. Sig. (2-sided)
Pearson Chi-Square	63.000(a)	56	.243
Likelihood Ratio	36.777	56	.978
Linear-by-Linear Association	5.640	1	.018
N of Valid Cases	9		

## Conclusion

(68.88%) of the 32 hospitals, 6 (64.95%) of the 9 public hospitals and 16 (69.64%) out of 23 private hospitals. Capacity indicators namely hospital equipment, hospital infrastructure, hospital surroundings, staff knowledge and staff capability were acceptable (above 60%) while inter hospital drills and inter hospital training were low. Drills and training are classified under the finance pillar of HS. Thus, it was deduced from the findings that probable inadequate financing towards inter hospital training and inter hospital drills could be the underlying cause of their low ratings in hospitals within Nairobi County. Study recommendations were that inter hospital training and inter hospital drills is strengthened for increased hospital capacity for disaster preparedness in Nairobi County and HSS.
